# Fungal soil communities in a young transgenic poplar plantation form a rich reservoir for fungal root communities

**DOI:** 10.1002/ece3.305

**Published:** 2012-07-12

**Authors:** L Danielsen, A Thürmer, P Meinicke, M Buée, E Morin, F Martin, G Pilate, R Daniel, A Polle, M Reich

**Affiliations:** 1 Department of Forest Botany and Tree Physiology, Büsgen-Institute, Georg-August University of GöttingenBüsgenweg 2, 37077, Göttingen, Germany; 2 Department of Genomic and Applied Microbiology & Göttingen Genomics Laboratory, Georg-August University of GöttingenGrisebachstr. 8, 37077, Göttingen, Germany; 3 Department of Bioinformatics, Georg-August University of GöttingenGoldschmidtstr. 1, 37077, Göttingen, Germany; 4 INRA de Nancy, UMR 1136 INRA/Nancy Université, Interactions Arbres/Microorganimes54280, Champenoux, France; 5 INRA, UR0588, Amélioration, Génétique, et Physiologie ForestièresCS 40001 Ardon, F-45075, Orléans Cedex 2; 6 University of Bremen, NW2/Plant PhysiologyLeobener Str. 2, G-28359, Bremen, Germany

**Keywords:** Community ecology, environmental DNA, fungi, genetically modified organisms, metagenomics, microbial biology

## Abstract

Fungal communities play a key role in ecosystem functioning. However, only little is known about their composition in plant roots and the soil of biomass plantations. The goal of this study was to analyze fungal biodiversity in their belowground habitats and to gain information on the strategies by which ectomycorrhizal (ECM) fungi form colonies. In a 2-year-old plantation, fungal communities in the soil and roots of three different poplar genotypes (*Populus* × *canescens*, wildtype and two transgenic lines with suppressed cinnamyl alcohol dehydrogenase activity) were analyzed by 454 pyrosequencing targeting the rDNA internal transcribed spacer 1 (ITS) region. The results were compared with the dynamics of the root-associated ECM community studied by morphotyping/Sanger sequencing in two subsequent years. Fungal species and family richness in the soil were surprisingly high in this simple plantation ecosystem, with 5944 operational taxonomic units (OTUs) and 186 described fungal families. These findings indicate the importance that fungal species are already available for colonization of plant roots (2399 OTUs and 115 families). The transgenic modification of poplar plants had no influence on fungal root or soil communities. Fungal families and OTUs were more evenly distributed in the soil than in roots, probably as a result of soil plowing before the establishment of the plantation. Saprophytic, pathogenic, and endophytic fungi were the dominating groups in soil, whereas ECMs were dominant in roots (87%). Arbuscular mycorrhizal diversity was higher in soil than in roots. Species richness of the root-associated ECM community, which was low compared with ECM fungi detected by 454 analyses, increased after 1 year. This increase was mainly caused by ECM fungal species already traced in the preceding year in roots. This result supports the priority concept that ECMs present on roots have a competitive advantage over soil-localized ECM fungi.

## Introduction

Anthropogenic activities can cause dramatic changes in ecosystem structures and their ecological services (Dawson 2011). Stability and maintenance of ecosystems rely on biodiversity and functional dynamics of organisms (Johnson et al. 1996). The impact of organismal groups on ecosystem stability depends on several factors such as adaptation strategies, interaction with other organisms (Johnson et al. 1996), and manner of nutrient acquisition. Fungi are a group of central importance as they play key roles in the carbon and nitrogen cycle improving the availability of nutrients for other organisms. They are distributed across all climatic zones, and colonize different habitats in ecosystems such as soil (Bridge and Spooner 2001), plant tissues (Arnold et al. 2000), water (Jones 2011), or rocks (Gadd 2007).

According to their lifestyle and ecological function, fungi can be classified to be saprophytic, pathogenic, endophytic, and mycorrhizal. Traditionally, those different groups have been analyzed separately by targeted approaches. With the advent of deep sequencing techniques, it is now possible to record these communities comprehensively as a precondition to understanding their interactions. For example, the analysis of rhizosphere and root endophyte communities in two natural poplar stands on contrasting soils revealed differentiation of the communities between roots and soil as habitats, but surprisingly, no significant soil-related effects (Gottel et al. 2011). Furthermore, in contrast to previous morphotyping/cloning studies in poplar plantations (Kaldorf et al. 2004; Stefani et al. 2009), deep sequencing suggested that mycorrhiza-forming fungal genera were underrepresented in roots (Gottel et al. 2011). It has been speculated that genetic differences between poplar species affect mycorrhizal colonization (Tagu et al. 2001; Karlinski et al. 2010), and thus influence the composition of fungal communities in roots (Gottel et al. 2011). Strong variation has been found among ectomycorrhizal (ECM) fungi that colonize specific coniferous species influenced by plant genotypes (Dučić et al. 2009; Karlinski et al. 2010). Many ECM fungi show strong host preferences (Lang et al. 2011), but the whole root-inhabiting fungal community is composed of different ecological groups. It is unknown whether fungal root communities as a whole can also be affected by the plant genotype.

Poplars are an important feedstock for biofuel production (Polle and Douglas 2010). Agro-forest areas are currently being expanded to meet the demand for sustainable biomass production. As soil-borne fungi have critical impact on plant health and productivity, the conservation of healthy communities of soil biota and biological soil management are considered pivotal to ensure soil fertility and overall productive and sustainable agricultural systems (Matson et al. 1997). However, knowledge of structure, function, and ecology of soil microbial communities is still very limited, especially for managed agro-forest plantations. As there is increasing interest in the use of fast growing tree species for production of second generation biofuel, attempts are underway to increase pulping properties of the wood by transgenic modification of lignin content and composition (Baucher et al. 1996; Pilate et al. 2002). Previous studies showed faster decomposition of leaf litter of poplars with suppressed activity of cinnamyl alcohol dehydrogenase (antisense CAD) than that of wildtype leaves (Pilate et al. 2002). It is currently unknown whether changes in tissue composition of transgenic poplar also influence the assemblage of root-inhabiting fungi or whether transgenic poplars affect the fungal community in the soil.

The main goal of the present study was a comprehensive analysis of fungal biota in soil and roots of wildtype and two antisense CAD poplar genotypes to test the hypothesis that the soil forms a large species-rich reservoir that leads to the differentiation of distinct fungal communities in wildtype and transgenic poplars. We conducted our study in a recently established experimental short rotation plantation of hybrid poplar (*Populus tremula* × *P. alba*, syn. *P*. × *canescens*) wildtype and transgenic lines. We applied 454 pyrosequencing analyses for in-depth characterization of fungal communities using the rDNA ITS1 region as marker gene. The role of soil as reservoir for root colonization was investigated (i) on the base of taxa composition in fungal soil and root communities, (ii) with respect to clustering of functional fungal groups in roots of different genotypes and adjacent soil, and (iii) with regard to temporal dynamics of ECM communities identified by morphotyping/sequencing techniques compared to 454 pyrosequencing.

## Materials and Methods

### Plant materials and study site

*Populus tremula* × *Populus alba* (female clone INRA #717-1B4) wildtype and transgenic lines with a modified lignin metabolism were multiplied by micropropagation (Leple et al. 1992). In June 2008, rooted plantlets were planted outdoors in a field trial (47°83′N, 1°91′E) nearby the INRA in Orleans, France, on sandy soil with flint. Climate is typical of the Loire Valley with oceanic tendencies, westerly dominant winds, average annual precipitation of 600 mm, and a mean annual temperature of 10.4°C. Natural flora is acidophilic and characteristic of poor soils, with oak, birch, chestnut, pine, and heather as prominent species belonging to the phytosociologic order *Quercetalia robori-petraeae*.

**Figure 1:**
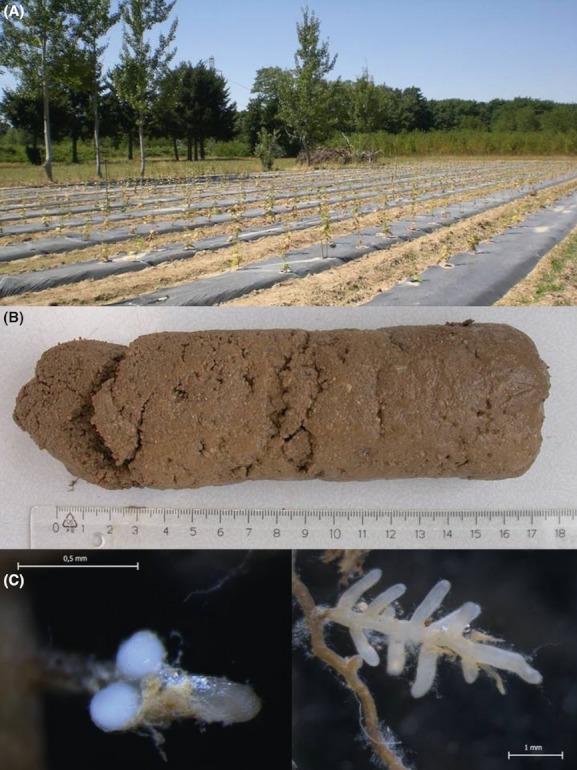
Soil cores (B) were taken on a 2-year-old poplar plantation (A) and cut into two longitudinal sections. 454 Pyrosequencing was applied on one half to study fungal soil and root communities. Out of the second half, poplar roots were isolated. Ectomycorrhizal fungi colonizing poplar roots were described by morphotyping (C) and ITS-sequencing (left picture: *Hebeloma sacchariolens*; right: *Laccaria tortilis*).

The field trial was established in an area of 1365 m^2^ with 120 plants per line (seven transgenic and one wildtype) (Fig. 1A). The plants were placed in randomized subplots, each consisting of 24 plants (four lines of six individual plants) (Supporting Information Fig. S1). The plants were drip irrigated during the growing period. In March 2010, all trees were coppiced according to typical management practices in a short rotation plantation.

### Sampling strategy

In October 2009, wildtype plants and two transgenic lines (ASCAD21 = L21, ASCAD52 = L18) with a decreased activity of CAD (Lapierre et al. 1999) were used for sampling. Three plots per line were chosen (Supporting information Fig. S1). In each plot, nine soil cores (depth: 0.2 m, diameter: 0.05 m) (Fig. 1B) were collected at a distance of 0.22 m between two neighboring poplar stems (for details, see Supporting Information Fig. S1). In total, we collected 81 soil cores (27 per poplar line). In addition, leaves were collected. Soil cores and leaves were transported on ice and processed in the laboratory within 72 h after sampling.

The soil cores (Fig. 1B) were cut longitudinally into two halves with a sterile scalpel (Supporting information Fig. S1). One half was used for analyses of ECM fungal community by morphotyping/ITS-sequencing and the other half for analyses of the overall fungal soil and root community by deep sequencing.

For ECM analyses three halves were pooled, resulting in three samples per plot. The samples were soaked in tap water and roots were removed by gentle washing. They were stored between wet filter papers at 4°C until further processing.

For analyses of 454 pyrosequencing, each sample was processed individually. Roots were cautiously removed from the soil, washed in autoclaved water, separated from roots of other plant species by shape and color under a stereomicroscope (Stemi SV 11, Zeiss, Jena, Germany) and frozen at –20°C. The soil was sieved, homogenized, subsampled in volumes of 2 mL, and stored at –20°C. Aliquots of the soil samples were used for nutrient element analyses.

### Soil analyses

Soil pH was determined after extraction in water for 4 h. Aliquots of the soil were weighed, dried for 4 days at 60°C, weighed again, and used to calculate the dry-to-fresh mass ratio. Carbon (C) and nitrogen (N) concentrations were determined by dry combustion using a C/N analyzer (Carlo Erbas Instruments, Italy). Mineral element concentrations of P, S, K, Ca, Mg, Mn, and Fe were determined using an Inductively Coupled Plasma – Atomic Emission Spectrometer (Spectro Flame, Spectro Analytic Instruments, Kleve, Germany) after pressure digestion of samples in 65% HNO_3_ for 12 h (Heinrichs et al. 1986). To determine the nitrate and ammonium concentrations, samples of 20 g soil were extracted in 40 mL 1 mmol/L CaCl_2_, filtered, freeze-dried, and dissolved in 0.5 mL double deionized water. The aliquots were used for photospectrometric measurement of nitrate and ammonium using commercial kits (Spectroquant, Merck, Darmstadt, Germany).

### DNA extraction and quality check

Eighty-one root samples and 10 leaf samples were freeze-dried and ground in a ball mill Type MM2 (Retsch, Haan, Germany). Hundred milligrams of root powder was suspended in 400 μL LSS-buffer of the “innuPREP Plant DNA kit” (analytikjena, Jena, Germany). Genomic DNA was extracted according to the manufacturer's instructions and eluted in 100 μL nuclease-free water (AppliChem, Darmstadt, Germany). Samples were checked for contamination by roots of other plant species by amplifying the trnL intron-region of the chloroplast DNA with the plant specific primer pair c (CGAAATCGGTAGACGCTACG) and d (GGGGATAGAGGGACTTGAAC) (Taberlet et al. 1991). The polymerase chain reaction (PCR) reaction mix was composed of 2 μL template DNA (up to 15 ng), 2.5 μL 10× buffer (Fermentas, St. Leon-Rot, Germany), 2 μL of MgCl_2_ (25 mmol/L, Fermentas), 1.25 μL of each primer (10 mmol/L) (Eurofins MWG Operon, Ebersberg, Germany), 0.5 μL dNTPs mix (10 mmol/L, Fermentas), 0.125 μL *Taq* polymerase (>10 U/μL, Fermentas), and 16.625 μL of nuclease-free water, resulting in a total volume of 25 μL. The PCR was performed in a Mastercycler Gradient (Eppendorf, Hamburg, Germany) starting with a hot-start at 95°C followed by 95°C for 1 min, 35 cycles of 30 sec at 94°C (denaturation), 30 sec at 53°C (annealing), and 1 min at 72°C (extension), and terminated with 72°C for 5 min. PCR products were subjected to electrophoresis in 2% agarose gels, ethidium bromide staining and were scanned (Raytest scanner FLA 5100, Straubenhardt, Germany). PCR products on the DNA of leaves of the same poplar lines as for roots were used as positive control. In the few cases where contamination was detected, new samples were prepared.

Eighty-one soil samples were dried in a SpeedVac-Concentrator Savant SPD 11V230 (Thermo, Bonn, Germany) and ground in a ball-mill. Genomic DNA was extracted using the Soil kit (MoBio, Carlsbad, CA) following the manufacturer's instructions.

### Amplicon generation and 454 pyrosequencing

All 162 DNA samples were amplified separately. Total extracted DNA was employed in the amplification at different concentrations (undiluted: 1:10, 1:50, 1:100). The Amplicon libraries were generated with primers including the Roche GS FLX Titanium Amplicon-Adaptor Sequences (A-Key, B-Key, Key: TCAG), a 10 bp multiplex identifier (MID1-29, see Table 1, TCB No. 005-2009, Roche, Mannheim, Germany) in front of the B-Adaptor for multiplexing the PCR products and the template-specific primers ITS1f (Gardes and Bruns 1993) and ITS2 (White et al. 1990), resulting in fusion primers A-ITS1F (5‘ CGTATCGCCTCCCTCGCGCCATCAG-CTTGGTCATTTAGAGGAAGTAA- 3‘) and B-MID-ITS2 (5‘ CTATGCGCCTTGCCAGCCCGCTCAG-MID-GCTGCGTTCTTCATCGATGC). PCR reactions were performed as described above, but 0.7 μL of 16 mg/mL bovine serum albumin (Merck, Darmstadt, Germany) was added to a total PCR mix volume of 25 μL. After amplification, the PCR products were purified using the “innuPREP PCRpure Kit” (analytikjena). Then, the PCR products from three cores of the same tree were pooled, resulting in 27 Amplicon libraries for root and soil, respectively, with independent replicates for each line. Amplicon concentration was determined using the Qubit™ dsDNA HS Assay Kit in a Qubit fluorometer (Invitrogen GmbH, Karlsruhe, Germany). The 27 amplicon libraries of root- and soil-samples, respectively, were pooled in equal amounts for 454 pyrosequencing. Amplicon libraries were sequenced with the 454 Genome Sequencer FLX (Roche) using the amplicon sequencing protocol and Titanium chemistry (Roche). Sequencing was performed by the Göttingen Genomics laboratory (http://www.g2l.bio.uni-goettingen.de/). Three medium lanes of a Titanium picotiter plate were used for sequencing of the complete amplicon libraries. The entire quality data set as unprocessed data files were deposited into the sequence read archive (SRA). The study accession number is ERP001442 and can be accessed by following link: http://www.ebi.ac.uk/ena/data/view/ERP001442.

**Table 1:** Summary of 454 pyrosequencing data

	Soil beneath		Roots from	
		
	Transgenic poplar	Wildtype poplar	Transgenic poplar	Wildtype poplars
Sequence reads	297,836	153,626	203,238	157,200
Sequence reads after quality control	251,883	129,962	166,556	137,652
Sequences/sample	11,631–15,965	9524–17,994	6568–10,835	4706–9620
Number of OTUs (nonsingletons/sample)	392–800	395–736	75–225	118–249
Number of singletons/sample	326–675	307–703	48–143	72–112

Samples are defined by sample type (soil or root samples) and poplar genotype (transgenic or wildtype). Twenty-seven samples were taken per sample type: Eighteen samples of transgenic and nine of wildtype plants, respectively. OTUs, operational taxonomic units

### Bioinformatics and OTU clustering

After the removal of barcodes and tags, 454 pyro sequencing reads were processed with a perl script discarding all reads shorter than 150 bp and reads containing more than four ambiguity symbols. On average, 74% of all reads passed these criteria. The individual sample FASTA files were subjected to cluster analysis for a tentative OTU count using the clustering function of USEARCH v5.3.23 (Edgar 2010) with the following criteria: ≥97% similarity over ≥90% sequence length. Cluster analyses were carried out on individual and “combined” samples, the latter ones including sequence read information of all soil or all root samples. All singletons were removed prior to further analyses. To identify OTUs at taxonomic level, a randomly selected sequence of an OTU cluster was compared with the nonredundant GenBank database (Benson et al. 2008) and the custom-curated database RSyst (http://mycor.nancy.inra.fr/RSyst/) using BLASTn (Altschul et al. 1997). A postprocessing perl script stored the 10 best BLASTn hits per cluster with an expectation value of <10e^−3^ in a BLASTn-file. OTUs with a taxonomic assignment at the species level were classified with respect to their ecological lifestyle by literature research (Table S3, Supplemental information). Ecological groups were categorized as follows: AM, arbuscular mycorrhizal; ECM, ectomycorrhizal; lichenized; saprotrophic; endophytic or parasitic.

### Morphotyping on root tips

Grass roots were identified by differences in morphology and removed. Three-hundred living root tips were inspected per poplar tree. ECM fungi were morphotyped (Fig. 1C) using a simplified method after Agerer (1987–2006) recording shape, color, texture of the mantle, and presence or absence of hyphae or rhizomorphes under a stereomicroscope (M205 FA, Leica, Wetzlar, Germany). ECM colonization (%) was calculated as: number of ECM root tips × 100/total number of root tips. Three to four ECM root tips of each morphotype were collected and stored at –20°C.

### Cloning and sequencing of ECM species

Genomic DNA of the frozen ECM root tips was extracted using the “innuPREP Plant DNA kit” (analytikjena). The rDNA ITS-region was amplified by PCR with the primer pair ITS5/ITS4 (White et al. 1990) as described above with the following modifications: 34 cycles and an annealing temperature of 55°C. Direct Sanger-sequencing or cloning/sequencing was carried out according to Lang et al. (2011). Sequences were blasted using the following databases: NCBI (nBLAST) (http://www.ncbi.nih.gov/), Fungal RSyst (http://mycor.nancy.inra.fr/RSyst/), and UNITE (http://unite.ut.ee/). Sequences are available at NCBI (accession JQ409279–JQ409296).

### Data analyses

To test for possible variability of fungal communities of different samples, 454 pyrosequencing data were blasted against the RSyst database. A perl script stored the top BLASTn hit (E-value < 10e^−3^) and the number of reads per species of each sample in a csv-file. Statistical analyses were performed on the basis of the number of reads per species and the relative abundance of reads. Samples were compared by a pairwise test based on the relative frequencies. The Wilcoxon rank-sum test was used to identify significant differences according to a *P*-value ≤ 0.05 after Bonferroni-correction. All our statistical analyses were carried out by using the software R-2.9.2 (R Development Core Team 2009). Additionally, nonmetric multidimensional scaling (NMDS) with the function metaMDS of the “vegan” package was applied. Before running the NMDS, data were square root transformed.

Statistical analyses of the fungal communities forming visible ECMs with roots were based on the relative abundance of the morphotypes. The Kruskal–Wallis rank sum test (package “stats”) was used to identify differences between poplar genotpyes (*P* ≤ 0.05).

The Wilcoxon rank sum test with an additional Bonferroni correction was carried out to examine differences in biodiversity indices and the relative abundances of fungal families within different sample types, respectively.

The defined OTUs were used to calculate taxon accumulation curves with the freeware software Analytic Rarefaction version 1.3 (http://www.uga.edu/strata/software/Software.html). Biodiversity indices and species richness estimators were calculated using the software EstimateS version 8.0.0 (Collwell 2006). Evenness was additionally determined by the formula (Shannon/LN [number of detected OTUs]).

Presence/absence data of fungal families in individual soil and root samples were subjected to hierarchical cluster analysis using EPCLUST (http://www.bioinf.ebc.ee/EP/EP/EPCLUST/index.cgi). Correlation-based distance measure was chosen as similarity metric and average distance as clustering method.

Differences in soil parameters were tested with one-way analysis of variance (ANOVA).

## Results

### Fungal species richness and diversity in soil and roots

In total, 811,900 sequence reads were generated by 454 pyrosequencing. Sequence reads that did not match our quality criteria were removed (see Material and Methods) resulting in 686,053 sequence reads for further analyses. In all, 4706–17,994 sequences were obtained per sample (Table 1). These sequences were clustered according to similarity and yielded 75–800 nonsingleton OTUs per sample (Table 1). Forty-eight to 703 singletons per sample were obtained (Table 1).

**Figure 2:**
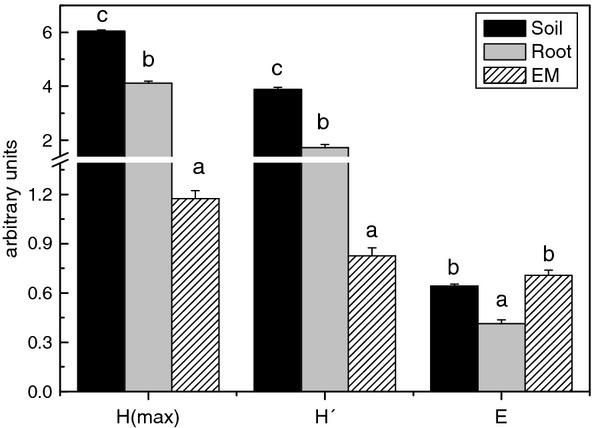
Species richness (H_max_), Shannon index (H′), and Evenness (E) of fungi in soil, roots, and of root-associated ECM communities. Diversity indices are means (*n* = 27 ± SE). Significant differences between bar heights (*P* ≤ 0.05) are indicated by different letters above bars. H_max_ = ln (species number).

Rarefaction curves based on 97% sequence identity leveled off between 398 and 817 OTUs for soil samples and between 91 and 249 OTUs for root samples (Supporting Information, Fig. S2A and B). Rarefaction analyses for complete fungal richness of the study site in soil and roots showed saturation at 5944 and 2399 OTUs, respectively (Supporting Information, Fig. S2D). Root samples exhibited higher variability in the shape of their species accumulation curves indicating strong scattering of species richness between different samples. Rarefaction analyses of ECM root communities revealed complete coverage (Supporting Information, Fig. S2C). Estimated species richness (H_max_) showed a clear decrease in the order of the habitats soil > roots > root-associated ECM communities (Fig. 2). The decrease in species richness from soil to roots and ECM communities was also reflected by the Shannon indices (Fig. 2). Evenness was highest for ECM communities and lowest for fungi in roots (Fig. 2).

### Fungal community structure in different habitats and poplar genotypes

To find out whether the poplar genotype affected fungal abundance or community structures in roots or soil, Wilcoxon rank-sum tests with Bonferroni correction were conducted. However, no significant difference was detected among the three investigated genotypes (transgenic lines ASCAD52 [=L18], ASCAD21 [=L21] and wildtype; *P* ≤ 0.05) with respect to the presence of fungal species or their abundance. These findings held true for soil and root samples as well as root-associated ECM fungi. Soil nutrients (per gram dry soil) did not differ between samples of different poplar genotypes (NO_3_^–^, 17.2 ± 2.04 μmol; NH_4_^+^, 15.3 ± 1.7 μmol; total N, 0.88 ± 0.08 mg; P, 0.22 ± 0.01 mg; S, 0.09 ± 0.01; Ca, 0.93 ± 0.04 mg; Mg, 0.51 ± 0.02 mg; Mn, 0.17 ± 0.01 mg; Fe, 4.16 ± 0.20 mg; C, 15.5 ± 1.4 mg; pH 5.85 ± 0.03), with the exception of K (mean: 1.19 ± 0.04 mg/g dry soil), which was slightly higher (13% above the mean) in soil collected beneath poplar line 18 than in that beneath the wildtype (*P* < 0.02).

**Figure 3:**
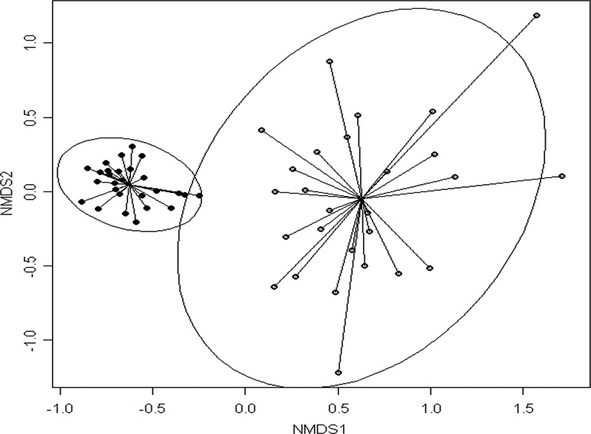
NMDS plot of the fungal community structure using the Bray–Curtis dissimilarity measure. Each point represents the fungal community of a given sample. Permutation tests revealed a highly significant classification (*P* = 0.001). Samples were classified according to the plant genotype (wildtype; L18 and L21, transgenic), sampling point, and sample type (black circles, soil; open circles, root). Stress value = 13.63, *R*^2^ = 0.98. Ellipses separate samples into two categories: left ellipse, soil samples; right one, root samples. Confidence area of ellipses = 0.95.

An NMDS plot calculated for OTUs revealed strong clustering of fungal communities for soil and roots, respectively (stress = 13.63, nonmetric fit *R*² = 0.98) (Fig. 3). Permutation test confirmed significant classification with *P* < 0.001 (*R*² = 0.6332). No separation of samples related to plant genotype or the position in the field was detected (Fig. 3). The significant differences between the fungal communities of soil and roots originated, therefore, from the lower species richness of roots compared with soil. Although soil contained higher species richness than roots, the scattering of data was lower (see ellipses in Fig. 3), indicating higher homogeneity of species distribution in soil than in roots.

### Fungal family abundance and distribution across soil and root samples

**Figure 4:**
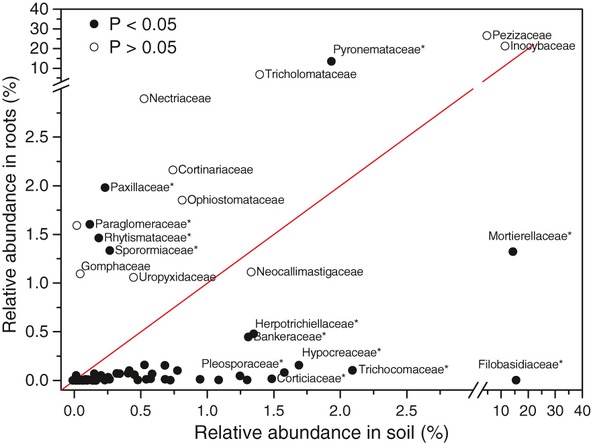
Distribution of fungal families in soil and root samples according to their relative abundance. Significant differences (*P* < 0.05) between soil and roots are indicated by black circles; open circles indicate fungal families with similar abundance in soil and roots. Families with abundances above 1% were labeled with a star. Red line indicates equal abundances in both roots and soil; 100% is the total abundance of all fungal families.

OTUs were clustered according to their taxonomic affiliation into overall 196 fungal families. Soil (186) and root (115) samples differed in their fungal family composition. Eighty-one fungal families were solely found in soil samples and 10 only in root samples (Supporting Information, Table S2). Among the common families, 59 were significantly more abundant in soil samples than in roots (Fig. 4). Two families, *Filobasidiaceae* and *Mortierellaceae,* were dominant in soil, each comprising about 15% of all OTUs. The relative abundance of nine further families in soil ranged between 1% and 5%, whereas all other fungal families that differed significantly from roots were present only with low abundance (<1%) (Fig. 4).

In root samples, six fungal families were significantly enriched in comparison with soil (Fig. S3). *Pyronemataceae* dominated the community (13.5%) in roots, while the relative abundances of *Paxillaceae*, *Paraglomeraceae*, *Rhytismataceae,* and *Sporormiaceae* ranged between 1.3% and 2%. *Russulaceae* were represented by 0.04% of the OTUs (Supporting Information, Table S1).

**Figure 5:**
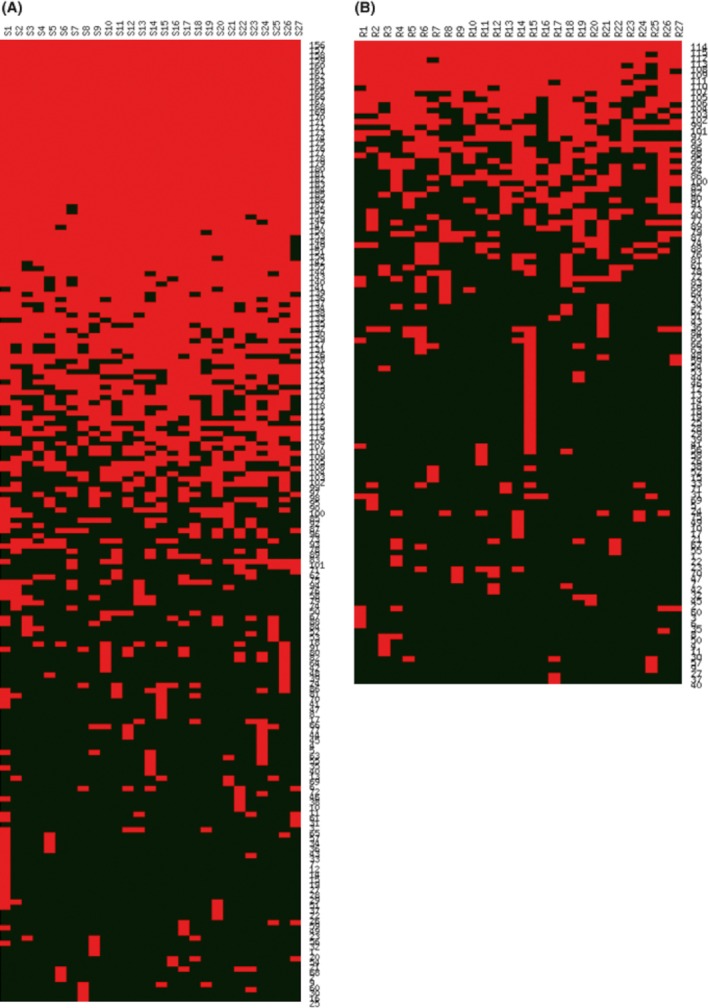
Heat map showing clustering of fungal families in (A) soil and (B) root samples. The color code of the heat map indicates presence (red) or absence (black) of fungal families (in rows) in the individual samples (in columns).

Hierarchical cluster analyses demonstrated the distribution pattern of fungal families in individual samples (Fig. 5). In soil samples, about one quarter of all fungal families were present in >90% of the samples. Forty-six percent and 59% of fungal families were detected in at least >50% and >25% of all soil samples, respectively (Fig. 5A). In contrast, the clustering of fungal families in root samples differed (Fig. 5B). Only 8% of all fungal families in root samples were present in >90% of all samples. Twenty percent and 38% of fungal families were present in >50% and >25% of the samples, respectively (Fig. 5B).

### Ecological groups in soil and root samples

**Figure 6:**
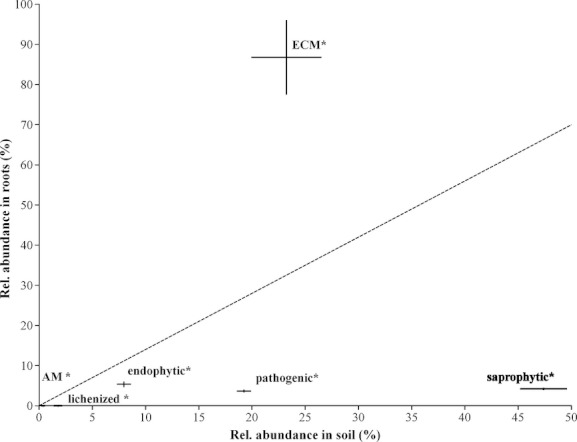
Distribution of fungal species with different ecological lifestyle in soil and root samples. OTUs with >97% sequence identity to known species were classified based on their taxonomic affiliation to six different ecological lifestyles. Only OTUs accounting for 90% of overall relative abundance were included in the analysis. All ecological lifestyle groups were significantly different (*P* < 0.05) from the dashed line, which indicates the same abundance in roots and soil. AM, arbuscular mycorrhizal; ECM, ectomycorrhizal fungal species; OTU, operational taxonomic unit.

To examine the distribution of ecological groups in soil and root samples 1272 and 463 OTUs, respectively, that could be assigned to species levels were selected and their abundances were set to 100%. One hundred and fifty-six and 27 of these species constituted 90% of the relative abundance in soil and roots, respectively, and were classified after literature research as ECM, AM, saprophytic, endophytic, pathogenic, or lichenized fungi (Supporting Information, Table S3). In soil samples, saprophytic fungi (47%) formed the largest group, followed by 23% ECM, 19% pathogenic, and 8% endophytic fungi. Lichenized and AM fungi were present only in low abundances of 1.8% and 0.4%, respectively (Fig. 6).

In root samples, ECM fungi were the dominant group encompassing 87% of the total abundance. Endophytic, parasitic, and saprophytic fungi showed lower abundances of 5%, 4%, and 4%, respectively. On the species level, no AM or lichenized fungi were detected (Fig. 6).

### Dynamic of the ECM community on poplar roots

With increasing age, poplar roots showed a typical increase in ECM fungal richness (Smith and Read 2008). In October 2009, seven, and in October 2010, nineteen ECM fungal species were detected on roots, of which six (2009) and 16 (2010) were identified by rDNA ITS sequencing (Supplemental information Table S4). With the exception of *Hebeloma* sp. and an uncultured Pezizales (DQ469743.1), the ECMs identified in 2009 were also present in 2010.

To understand dynamic processes within the ECM community and root colonization, morphotyping/ITS-sequencing and 454 pyrosequencing approaches were compared. All ECM species detected in 2009 were also detected by 454 pyrosequencing in both soil and root samples (Table 2). Furthermore, 13 of the 16 fungal species that colonized the roots in 2010 were already detected on poplar roots by 454 pyrosequencing in 2009. Two of the three missing species, *Scleroderma bovista,* and *Tuber* sp., were detected solely in soil samples. Only one species, an uncultured Ascomycota (EU557319.1) that formed an ECM in 2010, was detected neither in soil nor root samples in 2009 by 454 pyrosequencing approach.

**Table 2:** Fungal species detected by two approaches: morphotyping/ITS-sequencing and 454 pyrosequencing

	ITS-sequencing		454-Pyrosequencing	
		
	Ectomycorrhizal poplar root tips		Poplar roots	Soil
			
Fungal species	2009	2010	2009	2009
*Peziza ostracoderma*	x	x	x	x
*Paxillus involutus*	x	x	x	x
*Laccaria tortilis*	x	x	x	x
*Hebeloma sacchariolens*	x	x	x	x
*Tomentella ellisii*		x	x	x
*Scleroderma bovista*		x		x
*Cenococcum geophilum*		x	x	x
*Xerocomus ripariellus*		x	x	x
*Hebeloma* sp.	x		x	x
*Geopora* sp.				
*Tuber* sp.		x		x
Uncultured Ascomycota JQ409293		x		
Uncultured Ascomycota JQ409292		x	x	x
Uncultured ectomycorrhizal fungi JQ409294		x	x	x
Uncultured fungus JQ409288		x	x	x
Uncultured fungus JQ409287		x	x	x
Uncultured *Peziza* JQ409295		x	x	x
Uncultured Pezizales JQ409284	x		x	x

In October 2009 and 2010, ectomycorrhizal poplar root tips were sampled, classified by morphotyping and analyzed by ITS-sequencing (in total 27 samples). Additionally in 2009, poplar roots and soil samples were taken and subjected to 454 pyrosequencing analysis.

## Discussion

### Massive 454 pyrosequencing reveals surprisingly high fungal species richness in a young short rotation plantation

Rarefaction analyses indicated that we detected the majority of nonsingleton OTUs present in soil (average 556 per sample, 5944 OTUs for the complete survey) and roots (145 per sample, 2399 OTUs) of the complete experimental site of a 2-year-old poplar stand (Supporting Information, Fig. S2A and B). These numbers are relatively high compared with other studies reporting deep sequencing of fungal communities in soil of mature forest stands (Buee et al. 2009; Gottel et al. 2011) and roots from mature oak trees (Jumpponen et al. 2010). One reason may be a higher sampling density in our study compared with the previous ones. Nevertheless, it is remarkable that even in simple and young agro-ecosystems established on a tilled soil (Fig. 1A), very high sequencing depth is needed for comprehensive characterization of fungal community composition.

The fungal family richness (186 in soil and 115 in poplar roots) also exceeded values that have been previously reported for fungal soil communities (O'Brien et al. 2005; Buee et al. 2009), fungal phyllosphere (Jumpponen and Jones 2009), and root communities of oak (Jumpponen et al. 2010) (Supporting Information, Table S1). As no adjacent forest or mature site existed that could cause “vicinal invasion” (Kaldorf et al. 2004), our study shows that already very young stands own a rich and diverse reservoir of fungal propagules.

### Roots and soil constitute distinct ecological fungal biomes

We observed a clear separation of soil and root fungal communities (Fig. 3). A clear separation of microbiomes has also been reported for the rhizosphere and endosphere of mature poplar sites (Gottel et al. 2011). Our study shows that the differentiation of these habitats occurs already in an early phase of stand development and is mainly the result of fungal families enriched in soil (about 1/3 of all soil families) compared with roots. This observation points to high selectivity of interactions of roots with soil fungal genera (Fig. 3; Supporting Information, Table S1). The majority of significant fungal soil families shared saprophytic or parasitic lifestyles (Fig. 6) including the two most abundant fungal soil families, the *Filobasidiaceae* and *Mortierellaceae* (Hibbett et al. 2007). Members of these families are widespread, occurred also with high abundance in soils of six different tree mono-plantations and have therefore been classified as generalistic families (Buee et al. 2009).

Analysis of the lifestyle of the most abundant fungal species revealed significant enrichment of pathogenic, endophytic, lichenized, and AM fungi in soil compared with roots (Fig. 5). Some earlier studies demonstrated that pathogenic fungi are forming a large group within fungal communities in plant tissues (Monk and Samuels 1990; Bills and Polishook 1994) and that (bacterial) antagonists affect overall abundance of pathogenic fungi (Berg et al. 2002). However, the analysis of fungal communities in plant tissue samples has been challenging in the past due to inadequate isolation techniques (Bayman 2007). For example, in a deep sequencing study, Jumpponen et al. (2010) reported 12.3% of all detected fungi in mycorrhizal oak roots to be pathogenic. Our study shows that fungi with this lifestyle were about five-times more abundant in soil than in roots (Fig. 5).

Interestingly, the abundance of endophytic fungi was also higher in soil than in roots (Fig. 6). The mechanism of endophytic transmission is very variable and depends on the endophytic class (Rodriguez et al. 2009) ranging from spores dispersed by wind or rain to released hyphal fragments or infected (dead) plant tissue passively distributed by herbivores (Monk and Samuels 1990) or physical disturbance. These pathways and the influence of abiotic factors such as land-use lead to sometimes unexpected abundance and diversity of endophytic fungi (Rodriguez et al. 2009) found in different biomes such as agro-systems and terrestrial ecosystems (Arnold and Lutzoni 2007). Additionally, the identification of fungi as endophytes is problematic as the classification is often based on the momentary status of detection without regarding the future status of interaction (Schulz and Boyle 2005). Thus, fungi termed endophytic might be saprophytic or pathogenic in a certain part of their lifecycle.

Some distinct classes of mutualistic fungi including two families of mycorrhizal fungi (*Archaeosporaceae* [AM] and *Bankeraceae* [ECM]; Fig. 6, Supporting Information, Table S1) were significantly enriched in soil. The overabundance of AM fungi in soil is surprising as poplar trees are able to associate with both AM and ECM fungi at the same time (Molina et al. 1992). However, here ECM fungi formed the largest ecological group in roots with almost 90% abundance (Fig. 6) more than previously reported by Jumpponen et al. (2010) for ECM-colonized oak roots (72%). The strong colonization with ECM was probably caused by preceding long-term cultivation of poplars on the experimental sites and this may have suppressed AM proliferation (Dhillion 1994; Chen et al.^2000^).

The ECM accumulation in roots was mainly due to OTUs assigned to four families: *Inocybaceae, Pezizaceae, Paxillaceae*, and *Pyronemataceae* (Fig. 4). Whereas the former two were evenly distributed between soil and roots, the latter two were predominantly present in roots. Assignment of pezizalean *Pyronemataceae* taxa to specific ecological lifestyles remained problematic longtime, as they comprise a heterologous family. In fact, they are nowadays considered paraphyletic (Perry et al. 2007). In our study, *Pyronemataceae* showed significant presence in root samples (Fig. 4) and were one of the families with the highest genera richness (Supporting Information, Fig. S3). The different distribution of genera in soil and roots supports previously assigned ecological lifestyles of some taxa of the *Pyronemataceae*: in roots solely, genera described as mycorrhiza forming fungi were detected, while in soil, additional taxa with other ecological lifestyles were found.

The distribution of fungal families in individual samples was more homogeneous in soil than in roots (Fig. 5). This was also supported by the narrow clustering of OTUs in the NMDS analysis (Fig. 3) and the larger calculated Evenness in soil than in roots (Fig. 2). Mycorrhizal fungi are known to cluster along the root system of their host plants forming a patchy distribution (Smith and Read 2008). This may also be expected for fungal soil communities on early successional sites, as soil factors can differ widely at one site (Reverchon et al. 2010). In our study, the lack of significant differences in soil factors and soil plowing before the establishment of the plantation may have resulted in the relative homogeneous distribution of soil inhabiting fungi. The observation that a small number of ECM forming genera were dominant in roots and that roots contained a high number of rare OTUs at the same time suggests that roots were underlying high colonization pressure, but fungal proliferation was effectively suppressed with the exception of ECM. However, further studies are needed to shed light on the mechanisms influencing the composition of ecological groups in fungal communities in different habitats.

### Deep sequencing reveals host effects on the priority of ECM root colonization

The application of a double approach, morphotyping/Sanger-sequencing and 454-pyrosequencing, allowed us to draw a picture of dynamic processes and cross-links of fungal soil and root communities in relation to ECM colonization. The ECM community on poplar roots showed the well-known increases in colonization rate and diversity with increasing tree age (Dhillion 1994; Chen et al. 2000; Egerton-Warburton and Allen 2001). The fungal soil community (2009) harbored already all but one of the fungal species that formed ECM with poplar roots in the following year (2010, Table 2). This finding indicates the strength of fungal soil communities as a source for plant root colonization, and suggests low invasion by soil fungi from outside the agro-system within one annual cycle. Furthermore, most fungal species with ECM development in 2010 were already traced on poplar roots in 2009 by 454 pyrosequencing (Table 2). The experimental site was underlying early successional dynamics with factors that influence fungal root colonization such as the pattern of C allocation (Druebert et al. 2009), fungal competition (Kennedy et al. 2009), or availability of nutrients (Peter et al. 2001). While pronounced changes in soil nutrient availability appear unlikely, the growth of the poplars from about 0.2 to 1.9 m in the first year after planting (L. Danielsen, unpubl. results) indicates a strong increment in carbon productivity, which is one of the main drivers of ECM diversity (Druebert et al. 2009; Pena et al. 2010). The priority concept for ECM colonization, which has experimental support under controlled conditions (e.g., Kennedy et al. 2009), holds that the first mycorrhizal species to colonize a host's roots subsequently is the stronger competitor, when other fungal species are added. Our results suggest that this concept needs to be expanded to account for the dynamics of the colonized habitat. Most changes in ECM root communities were caused by fungal species already present on roots, that is, prior to other ECM present in soil that became more competitive forming functional ECM in the second year. As there were no changes in climatic or edaphic factors, which could have resulted in changes in the ECM assemblages, plant-related factors such as changes in carbon availability must have been responsible for the shift in the dominance of fungal species in the ECM communities.

### Transgenic poplars with suppressed CAD activity do not affect soil, root, or ECM communities

One important goal of this study was the assessment of the impact of transgenic versus wildtype poplar plants on fungal soil, root, and ECM communities; but no significant differences were observed (Fig. 3). Previous studies have already indicated no influence of transgenic poplar genotypes (rolC, a transformation causing stunting; npII::GUS, a selection marker coupled with a reporter gene) on ECM community structures (Kaldorf et al. 2004; Stefani et al. 2009). Here, we show that this also holds true for transgenic poplars (antisense CAD) with improved pulping properties that were modified in their phenylpropanoid metabolism (Pilate et al. 2002). This is an important result because other studies revealed significant correlations between genetically altered phenolic concentrations and associated above-ground organismic interactions (Kleemann et al. 2011). Earlier studies on genetically modified poplars were limited because only ECM or cultivable soil fungi could be analyzed. Our data add important information with regard to the bio-safety discussion because we show that in situ fungal soil and root communities were unaffected by host modification of an important commercial trait. These results are especially interesting where fungi are concerned that depend on host plant features, such as endophytic or parasitic fungi. In contrast to our working hypothesis, we did not detect any significant differences between the fungal communities of wildtype and antisense CAD poplars. Nevertheless, it is clear that genotype × biotic environment interactions cannot be excluded in general because intraspecific variations of ECM colonization have been demonstrated in crossing pedigrees (Labbé et al. 2011). Therefore, biotic interactions will have to be tested for each transgenic line that is planted in the field.

## Conclusions

The results of our analyses indicate that fungal soil and root community interact by dynamic processes and that soil is playing an important role as a fungal reservoir. Poplar roots were dominated by ECM fungi. The downregulation of an enzyme of lignin biosynthesis (antisenseCAD) did not affect ECM, root, or soil fungal assemblages. To our knowledge, we described for the first time the proportional composition of fungal ecological groups of two interacting fungal communities. Information on ecological groups and composition of fungal communities is urgently needed to understand the variable nature of fungal communities and underlying mechanisms of interaction. Additionally, the combination of two different detection techniques allowed us to draw a comprehensive picture of fungal soil and root communities of the experimental site.

## Data Accessibility

454 Pyrosequencing raw data set: SRA study accession number: ERP001442 (http://www.ebi.ac.uk/ena/data/view/ERP001442).

## Supporting Information

**Figure S1.** Scheme of the poplar plantation used for the field trial (A) and photo of a typical soil core (B).

**Figure S2.** Rarefaction curves of (A) soil, (B) root, (C) ECM root tip, and (D) combined samples.

**Figure S3.** Detected genera richness per fungal families clustered along their presence in soil and root samples.

**Table S1.** Relative abundance (mean ± SD) of the 198 fungal families detected by 454 pyrosequencing analysis in soil and root samples.

**Table S2.** List of fungal families used for cluster analyses (see Fig. 3).

**Table S3.** Fungal species were classified into different groups according to their ecological lifestyles. Literature source is indicated.

**Table S4.** Fungal species detected on ECM root tips of poplar plants by morphotyping/ITS-sequencing.
