# Supplementary material for: Fungal soil communities in a young transgenic poplar plantation form a rich reservoir for fungal root communities
Source: Ecol Evol. 2012 Jul 12;2(8):1935–48. doi: 10.1002/ece3.305 (PMC3433996; doi:10.1002/ece3.305)
Supplement: Supplementary file 1 [file ece30002-1935-SD1.docx]

# Supporting Information

**Figure S1: Scheme of the poplar plantation used for the field trial (A) and photo of a typical soil core (B).**

Within an area of 1365 m^2^, 840 poplar plants (*Populus tremula* x *Populus alba)* of seven different transgenic poplar lines (L5, L7, L9, L11, L18, L21, L22) and 120 wildtype plants (WT) were planted and organized in five different randomized replicates (1.-5. replicate). Each replicate consisted of seven plots with transgenic plants and one plot with wild type plants (6 x 4 plants per plot, black point representing one tree individual). The distance of the plants within one tree column in a plot was 1 m. The distance between the first two and the last two columns was 0.55 m whereas a distance of 1.5 m was kept between columns 2-3. Plots used for sampling are indicated by red circles. In each plot nine soil cores were collected at the positions indicated by the red stars. For further analyses, the soil cores were cut longitudinally and one half was used for classical EM analysis and the other for deep sequencing.


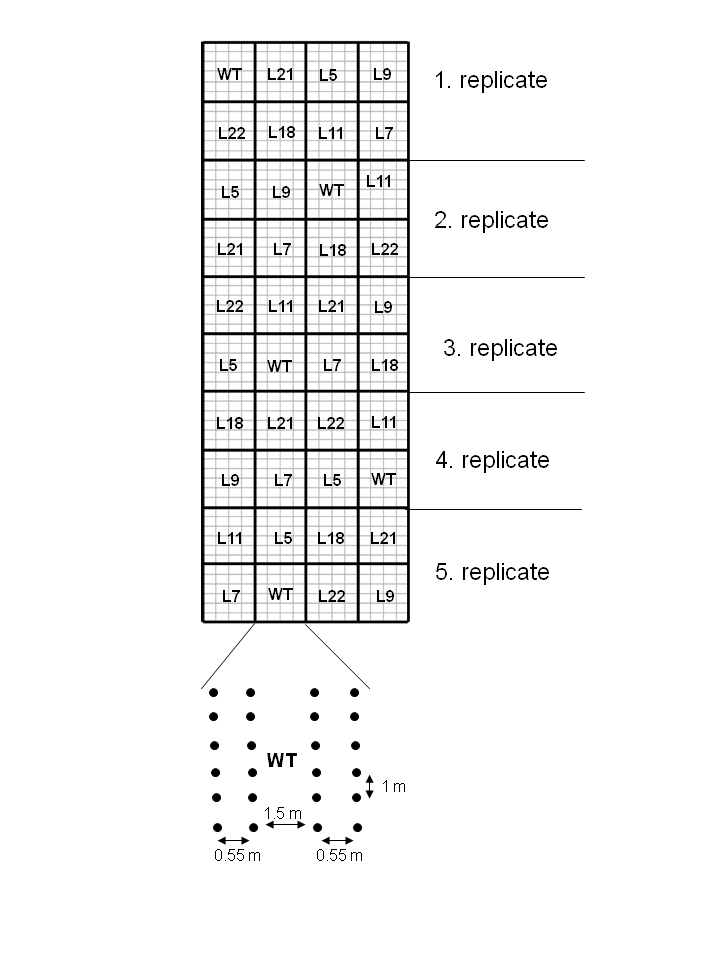

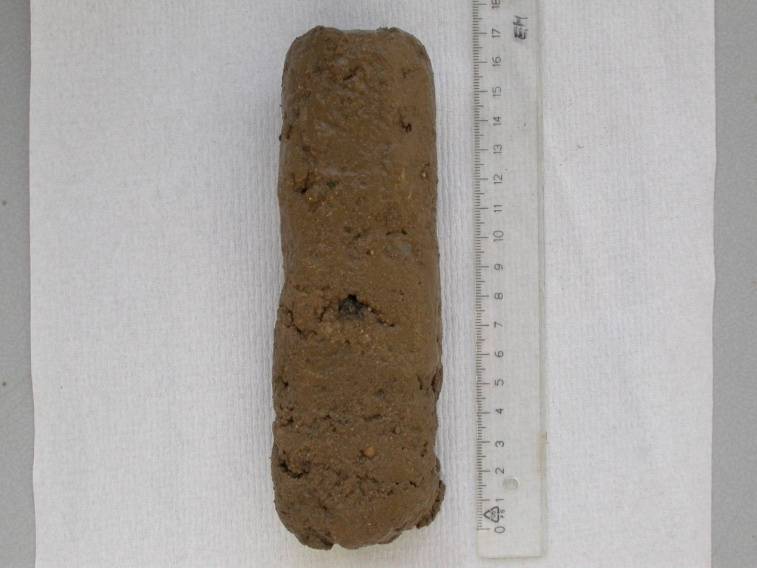


A

B
