# Supplementary material for: Fungal soil communities in a young transgenic poplar plantation form a rich reservoir for fungal root communities
Source: Ecol Evol. 2012 Jul 12;2(8):1935–48. doi: 10.1002/ece3.305 (PMC3433996; doi:10.1002/ece3.305)

**Figure S2: Rarefaction curves of (A) soil, (B) root, (C) ECM root tip and (D) combined samples.** For each individual sample a separate rarefaction curve was calculated (27 samples for soil and root samples, three for ECM root tip samples). Solid vertical line, for (A) and (B) mean of sequence reads ± standard deviation (SD) or for (C) mean of counted ECM root tips. Solid horizontal line, mean of counted OTU´s ± SD. Solid curves in (C) samples from 2009; dashed curves, samples from 2010. For (D) data of all soil and root samples were combined prior to cluster analysis, respectively. Solid curve in (D) combined soil samples; dashed curve, combined root samples.


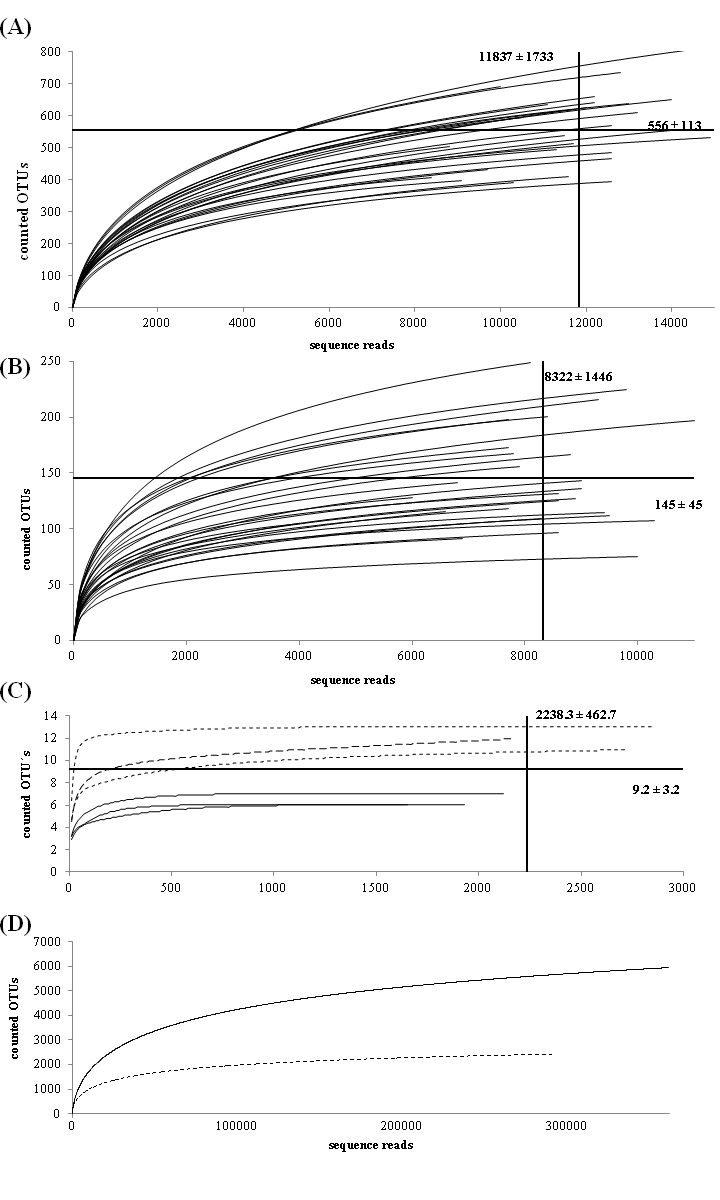

Supplement: Supplementary file 2 [file ece30002-1935-SD2.docx]
