# Supplementary material for: Fungal soil communities in a young transgenic poplar plantation form a rich reservoir for fungal root communities
Source: Ecol Evol. 2012 Jul 12;2(8):1935–48. doi: 10.1002/ece3.305 (PMC3433996; doi:10.1002/ece3.305)

**Figure S3: Detected genera richness per fungal families clustered along their presence in soil and root samples.** Dashed line, equal frequency in both sample types.


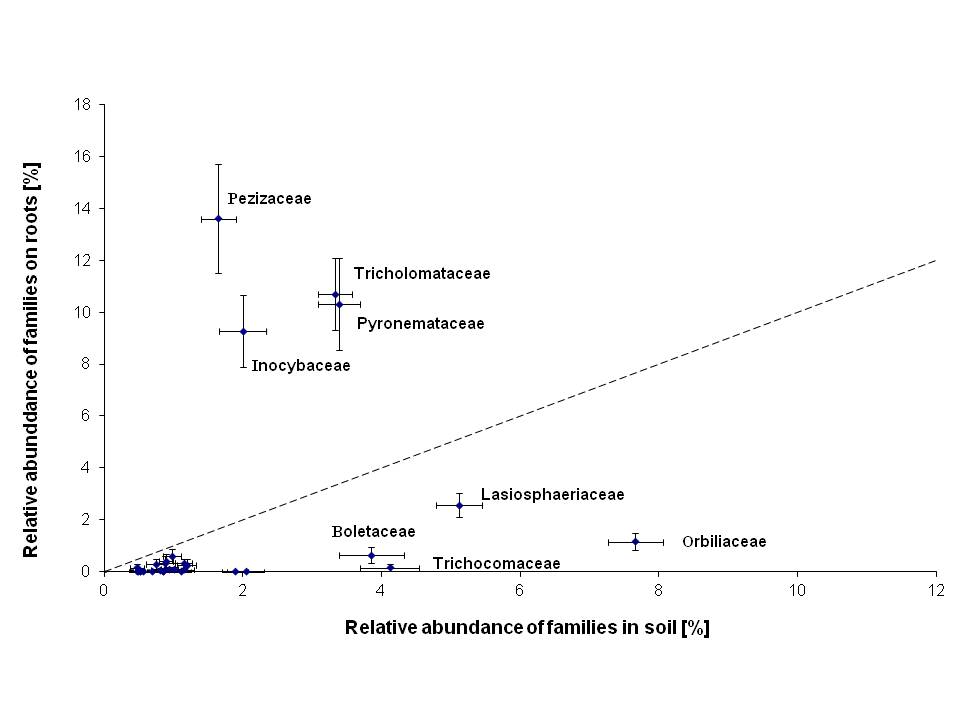

Supplement: Supplementary file 3 [file ece30002-1935-SD3.docx]
