# Supplementary material for: Fungal soil communities in a young transgenic poplar plantation form a rich reservoir for fungal root communities
Source: Ecol Evol. 2012 Jul 12;2(8):1935–48. doi: 10.1002/ece3.305 (PMC3433996; doi:10.1002/ece3.305)
Supplement: Supplementary file 4 [file ece30002-1935-SD4.docx]

**Table S1: Relative abundance (mean ± SD) of the 198 fungal families detected by 454 pyrosequencing analysis in soil and root samples.** Fifty-nine and six fungal families showed significant rel. abundance in soil and root samples, respectively (*, P ≤ 0.05).

|  | **Rel. Abundance (mean±SD)** | |
| --- | --- | --- |
| **Fungal Family** | **Soil Samples** | **Root Samples** |
| Acaulosporaceae | 0.01±0.04 | 0.04±0.10 |
| Acarosporaceae* | 0.19±0.31 | 0.02±0.06 |
| Agaricaceae* | 0.01±0.03 | 0.00±0.00 |
| Agyriaceae | 0.00±0.00 | 0±0 |
| Albatrellaceae* | 0.03±0.06 | 0.00±0.00 |
| Amanitaceae* | 0.02±0.04 | 0.00±0.00 |
| Amphisphaeriaceae* | 0.47±0.59 | 0.00±0.01 |
| Annulatascaceae | 0±0 | 0.00±0.00 |
| Archaeosporaceae* | 0.00±0.01 | 0±0 |
| Arthrodermataceae | 0.05±0.23 | 0±0 |
| Ascobolaceae | 0.00±0.02 | 0.00±0.00 |
| Astraeaceae | 0.00±0.00 | 0±0 |
| Atheliaceae* | 0.07±0.23 | 0.00±0.02 |
| Auriculariaceae | 0.00±0.00 | 0.01±0.04 |
| Auriscalpiaceae | 0.00±0.00 | 0±0 |
| Bankeraceae* | 1.30±2.75 | 0.44±2.23 |
| Basidiobolaceae* | 0.07±0.08 | 0.00±0.00 |
| Bionectriaceae* | 0.54±0.27 | 0.00±0.04 |
| Blastocladiaceae | 0.00±0.00 | 0±0 |
| Bolbitiaceae | 0.10±0.26 | 0.00±0.00 |
| Boletaceae* | 0.41±0.54 | 0.10±0.22 |
| Bondarzewiaceae | 0.00±0.00 | 0±0 |
| Botryobasidiaceae | 0.00±0.00 | 0.00±0.00 |
| Botryosphaeriaceae | 0.00±0.00 | 0±0 |
| Caliciaceae | 0.01±0.09 | 0±0 |
| Calosphaeriaceae | 0.00±0.01 | 0.00±0.00 |
| Candelariaceae | 0.00±0.02 | 0±0 |
| Cantharellaceae | 0.00±0.00 | 0.02±0.11 |
| Ceratobasidiaceae | 0.01±0.06 | 0±0 |
| Ceratocystidaceae | 0.00±0.00 | 0.00±0.00 |
| Chaetomiaceae* | 0.08±0.12 | 0.00±0.01 |
| Chaetosphaeriaceae* | 0.23±0.30 | 0.00±0.01 |
| Chionosphaeraceae | 0.00±0.00 | 0±0 |
| Choanephoraceae | 0.00±0.01 | 0±0 |
| Chytridiaceae* | 0.02±0.03 | 0±0 |
| Cladoniaceae | 0.00±0.00 | 0±0 |
| Clavariaceae | 0.00±0.00 | 0±0 |
| Clavicipitaceae* | 1.08±2.58 | 0.00±0.00 |
| Clavulinaceae | 0.00±0.00 | 0±0 |
| Coccotremataceae | 0±0 | 0.00±0.00 |
| Coniochaetaceae | 0.00±0.01 | 0±0 |
| Cordycipitaceae* | 0.09±0.15 | 0±0 |
| Coriolaceae* | 0.06±0.13 | 0±0 |
| Corticiaceae* | 1.48±2.10 | 0.01±0.05 |
| Cortinariaceae | 0.74±0.59 | 2.16±5.64 |
| Cunninghamellaceae | 0.00±0.00 | 0±0 |
| Cyphellaceae* | 0.05±0.20 | 0.00±0.00 |
| Cystofilobasidiaceae | 0.00±0.00 | 0±0 |
| Dacrymycetaceae | 0.00±0.00 | 0±0 |
| Davidiellaceae* | 0.34±0.26 | 0.06±0.26 |
| Debaryomycetaceae | 0.00±0.00 | 0±0 |
| Dermateaceae | 0.61±1.16 | 0.41±0.90 |
| Diatrypaceae | 0±0 | 0.00±0.03 |
| Didymellaceae* | 0.58±0.69 | 0.06±0.16 |
| Didymosphaeriaceae | 0.01±0.06 | 0±0 |
| Dipodascaceae* | 0.03±0.03 | 0±0 |
| Discinaceae | 0±0 | 0.00±0.00 |
| Dissoconiaceae | 0.00±0.00 | 0±0 |
| Dothioraceae* | 0.10±0.14 | 0.00±0.02 |
| Entolomataceae* | 0.13±0.27 | 0±0 |
| Entylomataceae | 0.00±0.00 | 0±0 |
| Eremomycetaceae* | 0.10±0.14 | 0.00±0.00 |
| Erysiphaceae | 0.00±0.00 | 0.01±0.08 |
| Erythrobasidiaceae | 0.00±0.00 | 0.01±0.06 |
| Exidiaceae | 0.03±0.03 | 0.35±0.85 |
| Filobasidiaceae* | 15.4±5.61 | 0.00±0.00 |
| Fistulinaceae | 0.02±0.08 | 0±0 |
| Ganodermataceae* | 0.01±0.01 | 0.00±0.04 |
| Gautieriaceae | 0.00±0.02 | 0±0 |
| Geoglossaceae | 0.00±0.01 | 0±0 |
| Gloeophyllaceae | 0±0 | 0.00±0.00 |
| Glomeraceae* | 0.31±0.20 | 0.07±0.13 |
| Glomerellaceae | 0.06±0.10 | 0.03±0.07 |
| Gomphaceae | 0.04±0.11 | 1.09±4.96 |
| Gomphidiaceae* | 0.57±0.56 | 0.01±0.05 |
| Haematommataceae | 0.01±0.06 | 0±0 |
| Halosphaeriaceae | 0.01±0.03 | 0.00±0.02 |
| Helotiaceae* | 0.45±0.84 | 0.05±0.11 |
| Helvellaceae | 0.00±0.00 | 0±0 |
| Hemiphacidiaceae | 0.07±0.16 | 0±0 |
| Herpotrichiellaceae* | 1.34±0.76 | 0.47±0.65 |
| Heterogastridiaceae | 0.00±0.01 | 0±0 |
| Hyaloscyphaceae | 0.01±0.02 | 1.59±3.33 |
| Hygrophoraceae | 0.00±0.02 | 0±0 |
| Hymenochaetaceae | 0.00±0.02 | 0±0 |
| Hymenogastraceae | 0.00±0.01 | 0±0 |
| Hypocreaceae* | 1.68±0.70 | 0.15±0.22 |
| Hyponectriaceae | 0±0 | 0.00±0.00 |
| Inocybaceae | 11.4±11.1 | 21.2±26.8 |
| Kickxellaceae | 0.00±0.00 | 0.00±0.00 |
| Lasiosphaeriaceae* | 0.77±0.55 | 0.09±0.17 |
| Lecanoraceae | 0.00±0.00 | 0.00±0.00 |
| Lecideaceae | 0.00±0.00 | 0±0 |
| Legeriomycetaceae | 0.00±0.00 | 0±0 |
| Lentinaceae | 0.00±0.00 | 0±0 |
| Leotiaceae | 0.04±0.07 | 0.06±0.22 |
| Leptosphaeriaceae* | 0.52±0.62 | 0.15±0.32 |
| Leucosporidiales | 0.09±0.14 | 0±0 |
| Lipomycetaceae* | 0.01±0.02 | 0±0 |
| Lobariaceae | 0.00±0.00 | 0±0 |
| Lophiostomataceae | 0.00±0.00 | 0±0 |
| Lycoperdaceae* | 0.72±2.09 | 0.00±0.00 |
| Lyophyllaceae | 0.00±0.00 | 0.00±0.00 |
| Magnaporthaceae | 0.11±0.44 | 0.73±1.45 |
| Malasseziaceae | 0.00±0.00 | 0±0 |
| Marasmiaceae | 0.00±0.00 | 0±0 |
| Massarinaceae* | 0.23±0.19 | 0.00±0.01 |
| Melanommataceae | 0.00±0.01 | 0±0 |
| Melanotaeniaceae | 0±0 | 0.03±0.14 |
| Meruliaceae | 0±0 | 0.00±0.00 |
| Metschnikowiaceae | 0.00±0.00 | 0±0 |
| Microascaceae | 0.00±0.00 | 0±0 |
| Microbotryaceae* | 0.15±0.18 | 0.06±0.35 |
| Monoblepharidaceae | 0.02±0.04 | 0±0 |
| Mortierellaceae* | 14.3±7.55 | 1.32±2.08 |
| Mucoraceae* | 0.16±0.15 | 0±0 |
| Mycosphaerellaceae | 0.07±0.07 | 0.30±0.67 |
| Myxotrichaceae | 0.03±0.15 | 0.00±0.00 |
| Nectriaceae | 0.52±0.57 | 2.89±3.23 |
| Neocallimastigaceae | 1.33±5.33 | 1.11±4.64 |
| Olpidiaceae* | 0.26±0.15 | 0.01±0.03 |
| Onygenaceae | 0.00±0.00 | 0±0 |
| Ophiocordycipitaceae | 0.10±0.19 | 0.03±0.14 |
| Ophiostomataceae | 0.81±0.57 | 1.85±3.15 |
| Orbiliaceae* | 1.24±1.15 | 0.04±0.07 |
| Pannariaceae | 0.00±0.01 | 0±0 |
| Paraglomeraceae* | 0.11±0.52 | 1.60±8.32 |
| Parmeliaceae | 0.00±0.00 | 0.00±0.00 |
| Paxillaceae* | 0.23±0.30 | 1.97±6.31 |
| Peniophoraceae | 0.00±0.01 | 0.01±0.07 |
| Pezizaceae | 4.76±6.00 | 26.5±27.2 |
| Phaeosphaeriaceae* | 0.68±0.71 | 0.15±0.42 |
| Phallaceae | 0.00±0.00 | 0±0 |
| Physalacriaceae | 0.03±0.15 | 0.00±0.01 |
| Physciaceae | 0.00±0.00 | 0.00±0.01 |
| Physodermataceae | 0.00±0.00 | 0±0 |
| Pilobolaceae | 0.00±0.00 | 0±0 |
| Pisolithaceae | 0.00±0.00 | 0.00±0.00 |
| Plectosphaerellaceae* | 0.25±0.26 | 0.03±0.09 |
| Pleosporaceae* | 1.57±1.20 | 0.07±0.17 |
| Pleurotaceae* | 0.68±0.64 | 0.00±0.03 |
| Podoscyphaceae | 0.01±0.03 | 0.00±0.00 |
| Polyporaceae* | 0.20±0.32 | 0.04±0.18 |
| Protomycetaceae | 0.00±0.00 | 0±0 |
| Psathyrellaceae | 0.27±0.53 | 0.17±0.39 |
| Pseudeurotiaceae* | 1.29±1.44 | 0.00±0.01 |
| Psoraceae* | 0.94±0.59 | 0.01±0.02 |
| Pucciniaceae | 0.00±0.00 | 0±0 |
| Pyronemataceae* | 1.93±2.07 | 13.5±13.5 |
| Rhizocarpaceae | 0.00±0.00 | 0±0 |
| Rhizophydiaceae* | 0.14±0.26 | 0±0 |
| Rhizopogonaceae* | 0.23±0.30 | 0.00±0.00 |
| Rhytismataceae* | 0.18±0.39 | 1.46±7.59 |
| Roccellaceae | 0.00±0.00 | 0±0 |
| Russulaceae* | 0.01±0.01 | 0.04±0.25 |
| Saccharomycetaceae | 0.04±0.17 | 0.00±0.00 |
| Saccharomycodaceae | 0.00±0.01 | 0±0 |
| Saccharomycopsidaceae | 0.00±0.00 | 0±0 |
| Sarcoscyphaceae | 0.15±0.72 | 0.00±0.03 |
| Schizophyllaceae | 0.00±0.00 | 0±0 |
| Schizosaccharomycetaceae | 0.00±0.00 | 0±0 |
| Sclerodermataceae | 0.01±0.06 | 0±0 |
| Sclerotiniaceae* | 0.40±0.54 | 0.07±0.30 |
| Scutellosporaceae | 0.00±0.01 | 0±0 |
| Sebacinaceae | 0.00±0.00 | 0.00±0.01 |
| Septobasidiaceae | 0.01±0.06 | 0±0 |
| Sordariaceae | 0.00±0.01 | 0±0 |
| Sphaerobolaceae | 0.00±0.00 | 0.00±0.02 |
| Spizellomycetaceae* | 0.15±0.25 | 0±0 |
| Sporormiaceae* | 0.26±0.33 | 1.33±6.91 |
| Stereocaulaceae | 0.00±0.00 | 0±0 |
| Strophariaceae* | 0.01±0.02 | 0.00±0.03 |
| Suillaceae | 0.02±0.13 | 0±0 |
| Sympoventuriaceae | 0.00±0.01 | 0.00±0.00 |
| Taphrinaceae | 0.03±0.07 | 0.00±0.00 |
| Tapinellaceae* | 0.02±0.02 | 0±0 |
| Teloschistaceae | 0.00±0.00 | 0±0 |
| Teratosphaeriaceae | 0±0 | 0.00±0.00 |
| Thelebolaceae* | 0.04±0.08 | 0.00±0.00 |
| Thelephoraceae | 0.21±0.28 | 0.75±2.07 |
| Trechisporaceae | 0.00±0.00 | 0.03±0.17 |
| Tremellaceae* | 0.02±0.02 | 0.00±0.03 |
| Trichocomaceae* | 2.09±1.76 | 0.10±0.32 |
| Tricholomataceae | 1.39±1.03 | 6.79±11.4 |
| Trichomonascaceae | 0.08±0.20 | 0.13±0.35 |
| Tuberaceae | 0.00±0.01 | 0±0 |
| Tubeufiaceae | 0.00±0.00 | 0±0 |
| Typhulaceae | 0.00±0.00 | 0±0 |
| Umbilicariaceae* | 0.08±0.10 | 0.01±0.03 |
| Uropyxidaceae | 0.44±1.34 | 1.05±2.90 |
| Ustilaginaceae | 0.00±0.00 | 0±0 |
| Venturiaceae | 0.00±0.00 | 0±0 |
| Verrucariaceae | 0.00±0.00 | 0±0 |
| Vuilleminiaceae | 0±0 | 0.00±0.00 |
| Xenasmataceae | 0.07±0.18 | 0.03±0.18 |
| Xylariaceae | 0.00±0.00 | 0±0 |
