# Supplementary material for: Fungal soil communities in a young transgenic poplar plantation form a rich reservoir for fungal root communities
Source: Ecol Evol. 2012 Jul 12;2(8):1935–48. doi: 10.1002/ece3.305 (PMC3433996; doi:10.1002/ece3.305)
Supplement: Supplementary file 5 [file ece30002-1935-SD5.docx]

**Table S2: List of fungal families used for cluster analyses (see Figure 3).** Listed in the descending order of appearance in heat map.

| **Clustering of Fungal Families in** | |
| --- | --- |
| **Soil Samples** | **Root Samples** |
| Acarosporaceae | Pezizaceae |
| Bionectriaceae | Pyronemataceae |
| Boletaceae | Inocybaceae |
| Clavicipitaceae | Nectriaceae |
| Cortinariaceae | Herpotrichiellaceae |
| Davidiellaceae | Mortierellaceae |
| Dermateaceae | Ophiostomataceae |
| Didymellaceae | Mycosphaerellaceae |
| Filobasidiaceae | Tricholomataceae |
| Glomeraceae | Magnaporthaceae |
| Herpotrichiellaceae | Nephromataceae |
| Hypocreaceae | Dermateaceae |
| Inocybaceae | Cortinariaceae |
| Lasiosphaeriaceae | Lasiosphaeriaceae |
| Leptosphaeriaceae | Glomeraceae |
| Microbotryaceae | Orbiliaceae |
| Mortierellaceae | Leptosphaeriaceae |
| Nectriaceae | Didymellaceae |
| Nephromataceae | Hypocreaceae |
| Ophiostomataceae | Phaeosphaeriaceae |
| Orbiliaceae | Pleosporaceae |
| Paxillaceae | Psathyrellaceae |
| Pezizaceae | Exidiaceae |
| Pleosporaceae | Bankeraceae |
| Pleurotaceae | Hyaloscyphaceae |
| Psathyrellaceae | Trichomonascaceae |
| Pseudeurotiaceae | Glomerellaceae |
| Psoraceae | Boletaceae |
| Pyronemataceae | Uropyxidaceae |
| Sporormiaceae | Leotiaceae |
| Trichocomaceae | Thelephoraceae |
| Tricholomataceae | Neocallimastigaceae |
| Amphisphaeriaceae | Helotiaceae |
| Phaeosphaeriaceae | Sporormiaceae |
| Eremomycetaceae | Paxillaceae |
| Helotiaceae | Umbilicariaceae |
| Mucoraceae | Trichocomaceae |
| Plectosphaerellaceae | Chaetosphaeriaceae |
| Lycoperdaceae | Davidiellaceae |
| Massarinaceae | Bionectriaceae |
| Olpidiaceae | Ophiocordycipitaceae |
| Polyporaceae | Acarosporaceae |
| Basidiobolaceae | Polyporaceae |
| Thelephoraceae | Pseudeurotiaceae |
| Umbilicariaceae | Gomphaceae |
| Mycosphaerellaceae | Halosphaeriaceae |
| Chaetosphaeriaceae | Rhytismataceae |
| Rhizopogonaceae | Clavicipitaceae |
| Bankeraceae | Sebacinaceae |
| Neocallimastigaceae | Bolbitiaceae |
| Rhizophydiaceae | Sphaerobolaceae |
| Corticiaceae | Psoraceae |
| Sclerotiniaceae | Gomphidiaceae |
| Cordycipitaceae | Melanotaeniaceae |
| Dothioraceae | Dothioraceae |
| Chaetomiaceae | Massarinaceae |
| Gomphidiaceae | Taphrinaceae |
| Dipodascaceae | Xenasmataceae |
| Entolomataceae | Vuilleminiaceae |
| Spizellomycetaceae | Calosphaeriaceae |
| Tapinellaceae | Erysiphaceae |
| Ophiocordycipitaceae | Cyphellaceae |
| Amanitaceae | Diatrypaceae |
| Ramalinaceae | Discinaceae |
| Exidiaceae | Erythrobasidiaceae |
| Glomerellaceae | Ganodermataceae |
| Rhytismataceae | Gloeophyllaceae |
| Thelebolaceae | Meruliaceae |
| Tremellaceae | Parmeliaceae |
| Russulaceae | Peniophoraceae |
| Strophariaceae | Teratosphaeriaceae |
| Albatrellaceae | Tremellaceae |
| Coriolaceae | Russulaceae |
| Lipomycetaceae | Sclerotiniaceae |
| Magnaporthaceae | Myxotrichaceae |
| Cyphellaceae | Sympoventuriaceae |
| Leotiaceae | Thelebolaceae |
| Atheliaceae | Eremomycetaceae |
| Chytridiaceae | Rhizopogonaceae |
| Trichomonascaceae | Pleurotaceae |
| Taphrinaceae | Podoscyphaceae |
| Uropyxidaceae | Corticiaceae |
| Ganodermataceae | Ascobolaceae |
| Paraglomeraceae | Lycoperdaceae |
| Agaricaceae | Acaulosporaceae |
| Archaeosporaceae | Microbotryaceae |
| Ascobolaceae | Chaetomiaceae |
| Myxotrichaceae | Filobasidiaceae |
| Sympoventuriaceae | Hyponectriaceae |
| Bolbitiaceae | Physalacriaceae |
| Venturiaceae | Olpidiaceae |
| Halosphaeriaceae | Agaricaceae |
| Onygenaceae | Kickxellaceae |
| Saccharomycodaceae | Lecanoraceae |
| Podoscyphaceae | Plectosphaerellaceae |
| Fistulinaceae | Lyophyllaceae |
| Melanommataceae | Basidiobolaceae |
| Hemiphacidiaceae | Amphisphaeriaceae |
| Acaulosporaceae | Ramalinaceae |
| Sarcoscyphaceae | Cantharellaceae |
| Kickxellaceae | Albatrellaceae |
| Hyaloscyphaceae | Auriculariaceae |
| Coniochaetaceae | Amanitaceae |
| Cladoniaceae | Atheliaceae |
| Septobasidiaceae | Saccharomycetaceae |
| Lyophyllaceae | Botryobasidiaceae |
| Sordariaceae | Physciaceae |
| Sclerodermataceae | Annulatascaceae |
| Arthrodermataceae | Coccotremataceae |
| Pisolithaceae | Pisolithaceae |
| Trechisporaceae | Sarcoscyphaceae |
| Schizosaccharomycetaceae | Ceratocystidaceae |
| Saccharomycetaceae | Paraglomeraceae |
| Monoblepharidaceae | Strophariaceae |
| Ceratocystidaceae | Trechisporaceae |
| Cystofilobasidiaceae |  |
| Malasseziaceae |  |
| Xenasmataceae |  |
| Bondarzewiaceae |  |
| Gomphaceae |  |
| Lecideaceae |  |
| Parmeliaceae |  |
| Stereocaulaceae |  |
| Lentinaceae |  |
| Rhizocarpaceae |  |
| Physalacriaceae |  |
| Erysiphaceae |  |
| Auriculariaceae |  |
| Pannariaceae |  |
| Sebacinaceae |  |
| Calosphaeriaceae |  |
| Lophiostomataceae |  |
| Pucciniaceae |  |
| Verrucariaceae |  |
| Pilobolaceae |  |
| Roccellaceae |  |
| Blastocladiaceae |  |
| Botryobasidiaceae |  |
| Clavulinaceae |  |
| Hygrophoraceae |  |
| Helvellaceae |  |
| Lobariaceae |  |
| Debaryomycetaceae |  |
| Xylariaceae |  |
| Botryosphaeriaceae |  |
| Erythrobasidiaceae |  |
| Scutellosporaceae |  |
| Lecanoraceae |  |
| Choanephoraceae |  |
| Clavariaceae |  |
| Cantharellaceae |  |
| Dacrymycetaceae |  |
| Auriscalpiaceae |  |
| Microascaceae |  |
| Peniophoraceae |  |
| Geoglossaceae |  |
| Heterogastridiaceae |  |
| Physciaceae |  |
| Entylomataceae |  |
| Caliciaceae |  |
| Cunninghamellaceae |  |
| Didymosphaeriaceae |  |
| Gautieriaceae |  |
| Marasmiaceae |  |
| Sphaerobolaceae |  |
| Tuberaceae |  |
| Tubeufiaceae |  |
| Ceratobasidiaceae |  |
| Hymenogastraceae |  |
| Physodermataceae |  |
| Schizophyllaceae |  |
| Typhulaceae |  |
| Teloschistaceae |  |
| Protomycetaceae |  |
| Hymenochaetaceae |  |
| Dissoconiaceae |  |
| Agyriaceae |  |
| Metschnikowiaceae |  |
| Haematommataceae |  |
| Phallaceae |  |
| Suillaceae |  |
| Astraeaceae |  |
| Candelariaceae |  |
| Ustilaginaceae |  |
| Chionosphaeraceae |  |
| Legeriomycetaceae |  |
| Saccharomycopsidaceae | |
