# Supplementary material for: Fungal soil communities in a young transgenic poplar plantation form a rich reservoir for fungal root communities
Source: Ecol Evol. 2012 Jul 12;2(8):1935–48. doi: 10.1002/ece3.305 (PMC3433996; doi:10.1002/ece3.305)
Supplement: Supplementary file 6 [file ece30002-1935-SD6.docx]

**Supplemental Information Table S3: Fungal species were classified into different groups according to their ecological lifestyles. Literature source is indicated.** Only species accounting for 90% of overall relative abundance were included in the analysis. (A) Fungal species detected in soil samples. (B) Fungal species detected in root samples. (C) Reference list.

**(A) Soil samples**

| **Species** | **Functional group** | **Source** |
| --- | --- | --- |
| *Acremonium furcatum* | endophyte | (Macia-Vicente et al. 2008) |
| *Aleuria aurentia* | saprophyte | (Rahi et al. 2009) |
| *Alternaria citri* | pathogen | (Isshiki et al. 2001) |
| *Alternaria longipes* | pathogen | (Stavely& Main 1970) |
| *Alternaria macrospora* | pathogen | (Bashi et al. 1983) |
| *Ampelomyces humuli* | pathogen | (Kiss 1997) |
| *Apodus deciduus* | saprophyte | (Malloch 1971) |
| *Apophysomyces elegans* | pathogen | (Lakshmi et al. 1993) |
| *Arthrobotrys amerospora* | pathogen | (Schenck et al. 1980) |
| *Arthrobotrys hertziana* | pathogen | (Nordbring-Hertz 2004) |
| *Arthrographis cuboidea* | pathogen | (Anagnost et al. 1994) |
| *Aspergillus versicolor* | pathogen | (Jussila et al. 2002) |
| *Athelia bombacina* | pathogen | www.mycobank.org |
| *Basidiobolus ranarum* | pathogen | (Zavasky et al. 1999) |
| *Bionectria ochroleuca* | saprophyte | (Ravnskov et al. 2006) |
| *Blastobotrys proliferans* | pathogen | (Quirin et al. 2007) |
| *Boletus dryophilus* | ECM | (Egerton-Warburton et al. 2007) |
| *Cenococcum geophilum* | ECM | (Jany et al. 2002) |
| *Cercophora sparsa* | saprophyte | (Hilber& Hilber 1979) |
| *Chaetosphaeria chloroconia* | saprophyte | (Midgley et al. 2002) |
| *Cheilymenia stercorea* | saprophyte | (Denison 1964) |
| *Chroogomphus rutilus* | ECM | www.deemy.de |
| *Cladophialophora chaetospira* | saprophyte | (Mouhamadou et al. 2011) |
| *Coniothyrium sporulosum* | pathogen | (Montecchio et al. 2004) |
| *Coprinopsis latispora* | saprophyte | (Prydiuk 2010) |
| *Cortinarius saturninus* | ECM | (Clemmensen & Mechelsen 2006) |
| *Cryptococcus podzolicus* | saprophyte | (Botes et al. 2005) |
| *Cryptococcus saitoi* | saprophyte | (Passoth et al. 2009) |
| *Cryptococcus terricola* | saprophyte | (Pedersen 1958) |
| *Cudoniella clavus* | saprophyte | (Dennis 1971) |
| *Cylindrocarpon olidum* | saprophyte | (Allegrucci et al. 2009) |
| *Discostroma tricellulare* | endophyte | (Okane et al. 1998) |
| *Drechslera biseptata* | pathogen | (Leach& Tulloch 1972) |
| *Entrophospora infrequens* | AM | (Vogelsang et al. 2006) |
| *Fusarium lateritium* | pathogen | (Hyun& Clark 1998) |
| *Fusarium oxysporum* | pathogen | (Allegrucci et al. 2009) |
| *Fusarium solani* | pathogen | (Woloshuk& Kolattukudy 1986) |
| *Fusarium solanifradicicola* | pathogen | (Suga et al. 2000) |
| *Glomus aurantium* | AM | (Blaszkowski et al. 2004) |
| *Handkea excipuliformis* | saprophyte | www.mycobank.org |
| *Hebeloma sacchariolens* | ECM | (Leski et al. 2010) |
| *Hyalodendriella betulae* | endophyte | (Crous et al. 2007a) |
| *Hypocrea viridescens* | pathogen | (Blaszczyk et al. 2011) |
| *Inocybe curvipes* | ECM | (Leski et al. 2010) |
| *Kabatiella zeae* | pathogen | (Pronczuk et al. 2004) |
| *Laccaria tortilis* | ECM | www.deemy.de |
| *Leccinum lepidum* | ECM | www.deemy.de |
| *Leptodontidium elatius* | pathogen | (Vasiliauskas et al. 2005) |
| *Leptodontidium orchidicola* | endophyte | (Fernando& Currah 1996) |
| *Leptosphaeria dryadis* | endophyte | (Promputtha et al. 2007) |
| *Leptosphaerulina chartarum* | pathogen | (Toth et al. 2007) |
| *Lirula macrospora* | pathogen | (Hennon 1990) |
| *Mariannaea elegans* | saprophyte | (Vasiliauskas et al. 2005) |
| *Metarhizium anisopliae* | pathogen | (Kershaw et al. 1999) |
| *Microbotryum stellariae* | pathogen | (Lutz et al. 2008) |
| *Mollisia cinerea* | endophyte | (Barklund& Kowalski 1996) |
| *Monacrosporium elegans* | pathogen | (Hao et al. 2004) |
| *Monacrosporium lobatum* | pathogen | (Li et al. 2005) |
| *Mortierella alpina* | saprophyte | (Kwasna et al. 2000) |
| *Mortierella elongata* | saprophyte | (Gams et al. 1972) |
| *Mortierella gamsii* | saprophyte | (Vasiliauskas et al. 2005) |
| *Mortierella horticola* | endophyte | (Holdenrieder& Sieber 1992) |
| *Mortierella humilis* | saprophyte | (Varnaite& Raudoniene 2005) |
| *Mortierella hyalina* | saprophyte | (Carreiro& Koske 1992) |
| *Mortierella verticillata* | endophyte | (Summerbell 2005) |
| *Nectria lugdunensis* | endophyte | (Seymour et al. 2004) |
| *Neofabraea alba* | pathogen | (Henriquez 2005) |
| *Neofabraea malicorticis* | pathogen | (de Jong et al. 2001) |
| *Neonectria ramulariae* | endophyte | (Shiono et al. 2008) |
| *Nolanea sericea* | saprophyte | www.mycobank.org |
| *Olpidium brassicae* | pathogen | (Teakle 1960) |
| *Ophiostoma floccosum* | pathogen | (Tanguay et al. 2006) |
| *Paxillus vernalis* | ECM | (Cripps 2003) |
| *Penicillium chrysogenum* | saprophyte | (Allegrucci et al. 2009) |
| *Penicillium concentricum* | saprophyte | (Samson et al. 1976) |
| *Peziza ostracoderma* | ECM | (Leski et al. 2010) |
| *Phaeosphaeria nodorum* | pathogen | (Keller et al. 1997) |
| *Phellodon niger* | ECM | www.deemy.de |
| *Phialocephala fortinii* | endophyte | (Grunig et al. 2002) |
| *Phialocephala xalapensis* | endophyte | (Grunig et al. 2002) |
| *Phialophora finlandia* | ECM | (Wilcox& Wang 1987) |
| *Phillipsia olivacea* | saprophyte | (Hansen et al. 1999) |
| *Phoma multirostrata* | endophyte | (Taylor et al. 1999) |
| *Phoma pomorum* | pathogen | (Conner et al. 2000) |
| *Pleopsidium discurrens* | lichen | (Obermayer 1996) |
| *Pleurotus cystidiosus* | saprophyte | (Cohen et al. 2002) |
| *Plicaria endocarpoides* | endophyte | (Hoffman et al. 2008) |
| *Podospora curvicolla* | saprophyte | (Wicklow& Yocom 1981) |
| *Podospora ellisiana* | saprophyte | (Angel& Wicklow 1983) |
| *Polyporus grammocephalus* | saprophyte | (Huang et al. 2011) |
| *Preussia africana* | endophyte | (Hoffman& Arnold 2010) |
| *Protoblastenia calva* | lichen | (Türk& Breuss 1994) |
| *Protoblastenia lilacina* | lichen | (Vezda 2008) |
| *Psathyrella hydrophila* | saprophyte | (Dix 1985) |
| *Pseudeurotium bakeri* | endophyte | (Tejesvi et al. 2011) |
| *Pseudeurotium ovale* | pathogen | (Willcox& Tribe 1974) |
| *Psora testacea* | lichen | (Papp et al. 1999) |
| *Pulvinula constellatio* | ECM | (Amicucci et al. 2001) |
| *Rhizopogon subbadius* | ECM | (Cripps& Grimme 2001) |
| *Rhizopus stolonifer* | pathogen | (Tian et al. 2002) |
| *Rhodotorula glutinis* | pathogen | (Davoli et al. 2004) |
| *Saccharicola bicolor* | pathogen | (Eriksson& Hawksworth 2003) |
| *Sclerotinia homoeocarpa* | pathogen | (Burpee 1997) |
| *Sistotrema sernanderi* | saprophyte | (Vasiliauskas et al. 2005) |
| *Sphaerosporella brunnea* | ECM | www.deemy.de |
| *Sporopachydermia quercuum* | endophyte | (Lachance et al. 1982) |
| *Stachybotrys echinata* | pathogen | www.mycobank.org |
| *Stachybotrys elegans* | pathogen | (Archambault et al. 1998) |
| *Stilbella byssiseda* | pathogen | www.mycobank.org |
| *Talaromyces ocotl* | saprophyte | (Heredia et al. 2001) |
| *Tetracladium maxilliforme* | endophyte | (Tedersoo et al. 2007) |
| *Tomentella ellisii* | ECM | (Cline et al. 2005) |
| *Tranzschelia fusca* | pathogen | (Maier et al. 2003) |
| *Trichocladium opacum* | saprophyte | (Allegrucci et al. 2009) |
| *Trichoderma aggressivum* | pathogen | (Savoie& Mata 2003) |
| *Trichoderma hamatum* | saprophyte | (Bae et al. 2009) |
| *Truncatella angustata* | pathogen | (Eken et al. 2009) |
| *Umbelopsis autotrophica* | saprophyte | (Renker et al. 2005) |
| *Umbelopsis isabellina* | saprophyte | (Kwasna et al. 2000) |
| *Vascellum pratense* | saprophyte | (Borovicka et al. 2005) |
| *Wilcoxina mikolae* | ECM | (Kernaghan et al. 2003) |
| *Xenasmatella vaga* | saprophyte | www.mycobank.org |
| *Xerocomus ripariellus* | ECM | (van der Heijden& Kuyper 2003) |
| *Zalerion varium* | saprophyte | (Ananda& Sridhar 2004) |
| *Zeloasperisporium hyphopodioides* | saprophyte | (Crous et al. 2007b) |

**(B) Root samples**

| **Species** | **Functional group** | **Source** |
| --- | --- | --- |
| *Clavariadelphus ligula* | ECM | (Smith et al. 2002) |
| *Dioszegia hungarica* | saprophyte | (Gacser et al. 2001) |
| *Flagelloscypha minutissima* | saprophyte | (Piatek& Cabala 2004) |
| *Hebeloma crustuliniforme* | ECM | www.deemy.de |
| *Hebeloma sacchariolens* | ECM | (Fox 1986) |
| *Inocybe curvipes* | ECM | (Leski et al. 2010) |
| *Laccaria tortilis* | ECM | www.deemy.de |
| *Lachnum pygmaeum* | endophyte | (Marquez et al. 2007) |
| *Leptodontidium orchidicola* | endophyte | (Wu& Guo 2008) |
| *Lirula macrospora* | pathogen | (Hennon 1990) |
| *Mortierella elongata* | saprophyte | (Gams et al. 1972) |
| *Nigrospora oryzae* | pathogen | (Wicklow& Poling 2009) |
| *Paxillus vernalis* | ECM | (Cripps 2003) |
| *Peziza ostracoderma* | ECM | (Leski et al. 2010) |
| *Phialocephala fortinii* | endophyte | (Grüning 2003) |
| *Phialophora finlandia* | ECM | (Wilcox& Wang 1987) |
| *Sphaerosporella brunnea* | ECM | www.deemy.de |
| *Terfezia boudieri* | ECM | (Zaretsky et al. 2006) |
| *Tomentella ellisii* | ECM | (Cline et al. 2005) |
| *Tranzschelia fusca* | pathogen | (Barnes& Szabo 2007) |
| *Wilcoxina mikolae* | ECM | (Kernaghan et al. 2003) |
