# Supplementary material for: Fungal soil communities in a young transgenic poplar plantation form a rich reservoir for fungal root communities
Source: Ecol Evol. 2012 Jul 12;2(8):1935–48. doi: 10.1002/ece3.305 (PMC3433996; doi:10.1002/ece3.305)
Supplement: Supplementary file 7 [file ece30002-1935-SD7.docx]

**Table S4: Fungal species detected on ECM root tips of poplar plants by morphotyping/ITS-sequencing.** Poplar roots were sampled in October 2009 and 2010.

Species ACC Best BLAST hit Source Source ACC Length of Homology Score

database fragment [%]

*Hebeloma sacchariolens* JQ409280 *Hebeloma sacchariolens* RSyst AY312985 460 97 850

*Hebeloma* sp. JQ409279 *Hebeloma* sp. UNITE UDB001188 605 96 957

*Laccaria tortilis* JQ409281 *Laccaria tortilis* UNITE UDB001589 568 99 1126

MT5 no sequence available

*Paxillus involutus* JQ409282 *Paxillus involutus* RSyst EU078741 638 99 1203

*Peziza ostracoderma* JQ409283 *Peziza ostracoderma* NCBI EU819461.1 657 99 1158

uncultured Pezizales JQ409284 uncultured Pezizales NCBI DQ469743.1 669 98 1112

*Cenococcum geophilum* JQ409285 *Cenococcum geophilum* NCBI HQ406817.1 857 96 1375

Geopora sp. JQ409286 *Geopora* sp. TAA 192232 NCBI FM206420.1 489 99 878

MT13 no sequence available

Uncultured fungi (Ascomycota) JQ409287 uncultured fungus NCBI EU555000.1 510 100 942

Uncultured fungi (Ascomycota) JQ409288 uncultured fungus NCBI EU554730.1 539 100 996

MT30 no sequence available

MT33 no sequence available

*Scleroderma bovista* JQ409289 *Scleroderma bovista* UNITE UDB002179 630 98 1205

*Tomentella ellisii* JQ409290 *Tomentella ellisii* NCBI DQ068971.1 504 100 931

*Tuber* sp. JQ409291 *Tuber* sp. GMB-2010b NCBI HM485376.1 473 100 874

uncultured Ascomycota JQ409292 uncultured Ascomycota NCBI EU562601.1 522 97 883

uncultured Ascomycota JQ409293 uncultured Ascomycota NCBI EU557319.1 544 99 992

uncultured ectomycorrhizal JQ409294 uncultured ectomycorrhizal NCBI EF484931.1 571 97 965

fungus fungus

uncultured *Peziza* JQ409295 uncultured Peziza NCBI GU969261.1 539 99 979

*Xerocomus ripariellus* JQ409296 *Xerocomus ripariellus*  UNITE UDB000485 649 100 1287
